# Promoting healthy and sustainable diets through food service interventions in university settings: a scoping review

**DOI:** 10.1186/s40795-025-01158-3

**Published:** 2025-09-15

**Authors:** Suzie Kratzer, Melissa A. Theurich, Theresa Mareis, Simone Proebstl, Nicole Holliday, Sebrina Yan, Anna Leibinger, Ina Monsef, Leonie Bach, Daniela Rincón Camargo, Lukas Schwingshackl, Aline Simonetti, Monika Hartmann, Dominic Lemken, Peter von Philipsborn

**Affiliations:** 1 Chair of Public Health and Health Services Research, Ludwig-Maximilians- Universität München (LMU Munich), Munich, Germany; 2 Pettenkofer School of Public Health, Munich, Germany; 3 Institute of Public Health, Faculty of Medicine and University Hospital Cologne, University of Cologne, Cologne, Germany; 4 Department of Agricultural and Food Market Research, Institute for Food and Resource Economics, University of Bonn, Bonn, Germany; 5 Institute for Evidence in Medicine, Medical Centre, Faculty of Medicine, University of Freiburg, University of Freiburg, Freiburg, Germany; 6 Department for Socioeconomics of Sustainable Nutrition, Institute for Food and Resource Economics, University of Bonn, Bonn, Germany; 7 Chair of Public Health Nutrition, University of Bayreuth, Universitätsstrasse 30, 95447 Bayreuth, Germany

**Keywords:** Healthy and sustainable diets, Canteens, Cafeterias, Dining halls, Universities, Food service, Food environments, Scoping review

## Abstract

**Background:**

Food service operations in universities and colleges may support healthy and sustainable diets among students and staff, thereby contributing to the transformation of the wider food system. Although numerous studies on relevant interventions have been conducted, no comprehensive and up-to-date review exists.

**Methods:**

We conducted a scoping review in line with the PRISMA-ScR guidelines. We included any study examining interventions in university or college food service settings aimed at, or potentially suitable for: (1) supporting healthy and/or sustainable diets, (2) reducing food waste, or (3) otherwise improving the sustainability of food service operations. We comprehensively searched six academic databases and conducted forward and backward citation searches. We considered studies using any systematic empirical study design, and extracted and charted data on key study characteristics.

**Results:**

We identified 206 studies reporting on 273 interventions. Most studies (69%) used quasi-experimental study designs and were conducted in North America (53%) or Europe (34%). The most common intervention approaches were labelling (34%), increasing the availability of healthy and/or sustainable foods (33%), and information and awareness-raising campaigns (22%). The most frequently assessed outcomes included implementation-related measures (e.g., costs, feasibility, acceptability), diet-related metrics (e.g., sales or consumption of specific foods), and sustainability indicators (e.g., carbon footprint). The majority of studies were short-term, with a median follow-up of 4 weeks.

**Conclusions:**

Multiple approaches for promoting health and sustainability in university and college food service settings exist, including improved offerings of healthy and sustainable foods, as well as labelling and educational interventions. Future studies should address existing evidence gaps and limitations by assessing both health and sustainability outcomes, and by using more robust study designs, and extending follow-up periods.

**Protocol registration:**

Registered prospectively with the Open Science Framework at 10.17605/OSF.IO/CM8VA.

**Supplementary Information:**

The online version contains supplementary material available at 10.1186/s40795-025-01158-3.

## Background

The global food system faces considerable challenges in terms of human health and environmental sustainability [1]. Suboptimal dietary patterns are among the leading risk factors for chronic disease and premature death worldwide [2, 3]. In addition, the global food system is responsible for a quarter to a third of global anthropogenic greenhouse gas emissions, thus contributing substantially to climate change [1, 4]. The food system is also the primary driver of further processes of global environmental change, including biodiversity and habitat loss, the depletion of freshwater resources, deforestation, and land degradation [1]. At the same time, approximately one-third of global food production is estimated to be lost or wasted [5]. A shift towards healthier and more sustainable dietary patterns is therefore crucial to safeguard human and planetary health, as is a reduction in food waste and a move to more sustainable production practices along the food supply chain [1].

Institutions of tertiary education (universities, colleges, etc.) can address these challenges in various ways, including through their core functions of education, research, and societal outreach [6, 7]. A further avenue for making an impact is through campus food services, such as cafeterias, canteens, kiosks, and cafés [8, 9]. University faculty, staff, and students generally use campus food services. Students and staff often spend a substantial part of their time on campus, where they often consume a considerable part of their diet. Students are typically young adults, transitioning from youth to more independent lifestyles in adulthood. This period has been shown to be a critical phase, during which changes in dietary habits often occur, that may last for the rest of adulthood [10]. Exposure to canteen foods has also been linked to dietary patterns that extend beyond the university and college setting [11]. Moreover, university food services may act as exemplars for food services in other settings, such as schools (including high schools) and workplaces. Exposure to healthy and sustainable diets on campus may also influence students’ expectations of food service later in life, whether in the workplace or as parents in school settings. Interest in sustainable diets is growing among younger generations in many high-income countries [12, 13], and positive experiences with healthy and sustainable campus food services may enable and empower students to act as change agents for a wider food system transformation [9]. Interventions to support healthy and sustainable diets in institutions of tertiary education may also yield more immediate positive effects. In particular, studies have shown that improving students’ diets can enhance their physical and mental health as well as overall well-being [14–16].

University food services are, therefore, a potentially important setting for interventions to promote health and sustainability. A substantial number of studies have been conducted on such interventions. However, to the authors’ best knowledge, no comprehensive, up-to-date review of the research landscape on this topic exists. To identify potential research gaps and to guide future research and practice in the field, this scoping review therefore aims to provide an overview of interventions implemented in university and college food service settings that aim (1) to support healthy and/or sustainable diets, (2) to reduce food waste, or (3) to otherwise improve the sustainability of food service operations (e.g. through improved energy efficiency).

## Methods

### Overview

>We used state-of-the-art scoping review methodology as recommended by the Joanna Briggs Institute (JBI) and the PRISMA-ScR reporting guideline (see Table s1 in supplementary file 1 for the PRISMA-ScR checklist) [17, 18]. The protocol for this review was registered and published with the Open Science Framework (OSF) at 10.17605/OSF.IO/CM8VA before conducting the literature search [19].

### Eligibility criteria

We included studies that fulfilled the following eligibility criteria:

- **Population**: Any person using food services on university or college campuses, as well as relevant stakeholders like canteen staff, managers, and suppliers.
- **Intervention**: Any intervention aimed at, or potentially suitable for promoting healthy and/or sustainable diets, reducing food waste, or otherwise increasing the environmental sustainability of food service operations, based on the definition of sustainability used by the authors of included studies. We included studies focusing on either health or environmental sustainability, or both.
- **Outcomes**: Any outcome falling into one or several of the following categories (studies had to report at least one of these outcomes to be eligible for inclusion):

i. Diet quality (e.g., food and meal choices, food and nutrient intake of canteen users).
ii. Health outcomes (e.g., body weight, Body Mass Index (BMI), prevalence and incidence of diet-related health outcomes, and mental health outcomes such as disordered eating and body image disorders).
iii. Sustainability outcomes (e.g., level of greenhouse gas emissions, level of animal-derived protein intake, amount of food waste, water usage).
iv. Implementation-related outcomes (e.g., feasibility, acceptability, costs, personnel needs).

- **Comparison**: No, or alternative interventions or business as usual.
- **Setting**: Any food service operation in universities, colleges, and similar institutions of tertiary education. This includes canteens, cafeterias, and dining halls, as well as kiosks or vending machines on campus. Studies on privately operated restaurants or cafés located on campuses were not included.
- **Study design**: Any systematic empirical study design (including both quantiative and qualitative studies) except reviews. Commentaries, letters, and similar publications not reporting on a systematic investigation were not included.
- **Publication type**: Any form of academic literature (peer-reviewed or non-peer-reviewed, including preprints) as well as grey literature. We did, however, not search specifically for preprints or grey literature.
- **Language**: We did not set any a priori language restrictions, but we conducted the searches in English only. Within our team, we were able to cover studies written in English, French, German, Spanish, and Portuguese.
- **Publication date**: No a priori publication date restrictions were set, and databases were searched from their onset.

We included mental health outcomes, as interventions to promote healthy and sustainable diets may have both positive and negative mental health impacts, including on outcomes such as weights concerncs and body image disorders.

### Search methods for identification of studies

We systematically searched six academic databases (MEDLINE, the Cochrane Library, Scopus, Epistemonikos, CAB Abstracts, and ERIC) and used references of existing reviews and key primary studies for forward and backward citation searches (i.e. snowballing searches) in Scopus. The search syntax for the database searches was based on three search concepts: (i) food service; (ii) tertiary education settings; and (iii) diet, health, or sustainability. The full search strategies for all databases and the references used for the snowballing searches are shown in supplementary file 1. The literature searches were designed and conducted by an information specialist (IM). Searches were conducted on June 8, 2023. We used Endnote for de-duplication.

### Title and abstract screening

We used the web-based application Rayyan for title and abstract screening [20]. References were screened by one review author (SK, TM, SP, MT, NH, AL, or AS). Only studies that were clearly irrelevant were excluded, while those marked as unclear were screened in duplicate by a second review author (SK or PvP). For references identified through MEDLINE, Scopus, and snowballing searches (constituting 38% of all references), we conducted a full manual screening, as we considered these databases to be particularly relevant. For references identified through CAB Abstracts, Epistemonikos, Cochrane Library, and ERIC, we restricted manual screening to references with a high to medium predicted relevance based on Rayyan’s prediction algorithm, excluding references with a star rating below 2.5 out of 5. Rayyan’s prediction algorithm calculates the likelihood that references will be eligible for inclusion based on prior manual screening decisions using artificial intelligence technology. The algorithm has been validated and shown to be reliable [21–23]. One validation study found that after screening 20% of all references manually, a star rating of 2.5 or higher had a sensitivity of 100% for identifying eligible studies, meaning that exclusion of references with a star rating below this threshold did not exclude any eligible studies [24]. As an additional validation measure, we manually screened 200 abstracts with a star rating of below 2.5, and did not identify any relevant study among these.

### Full text screening

Full texts were screened by one review author (SK, TM, AS, SP, NH, LB, or AL), and unclear cases were screened in duplicate by a second review author (SK or PvP). The reasons for excluding studies during the full-text stage were documented.

### Data extraction

Data extraction was done by one review author (SK, NH, SP, or TM) using the data extraction form shown in supplementary file 1. Any uncertainties were marked by the reviewer and double-checked by a second review author (SK or PvP). For 10% of all studies, all extracted data were double-checked by a second review author (PvP). We extracted data on study design, setting, intervention characteristics, outcomes, and the reported direction of effect.

### Data synthesis

We summarized results narratively, in tables, and graphically in an Evidence Gap Map. An Evidence Gap Map is a visual tool designed to provide an overview of the existing evidence on a topic, highlighting gaps in the evidence base while also showing where evidence is more abundant [25]. We used the NOURISHING Framework, developed by the World Cancer Research Fund International, to classify interventions [26].

### Quality assurance measures

We implemented several quality assurance measures. Among others, we piloted the procedures at each stage of the review process, conducted regular team meetings, drafted internal guidance documents, and kept a list of rolling questions that were updated throughout the review process to ensure clarity and consistency between review authors. As a calibration exercise, review authors involved in the screening (SK, TM, AL, NH, SP, AS, and PvP) independently assessed a set of references (50 at title and abstract stage, and 10 at full-text screening stage). Following this, inconsistencies and any questions that arose were discussed with the team, and the guidance documents were adjusted accordingly. The data extraction sheet was pilot-tested by two review authors (SK, PvP) on five studies.

## Results

### Results of the search and selection process

**Fig. 1:**
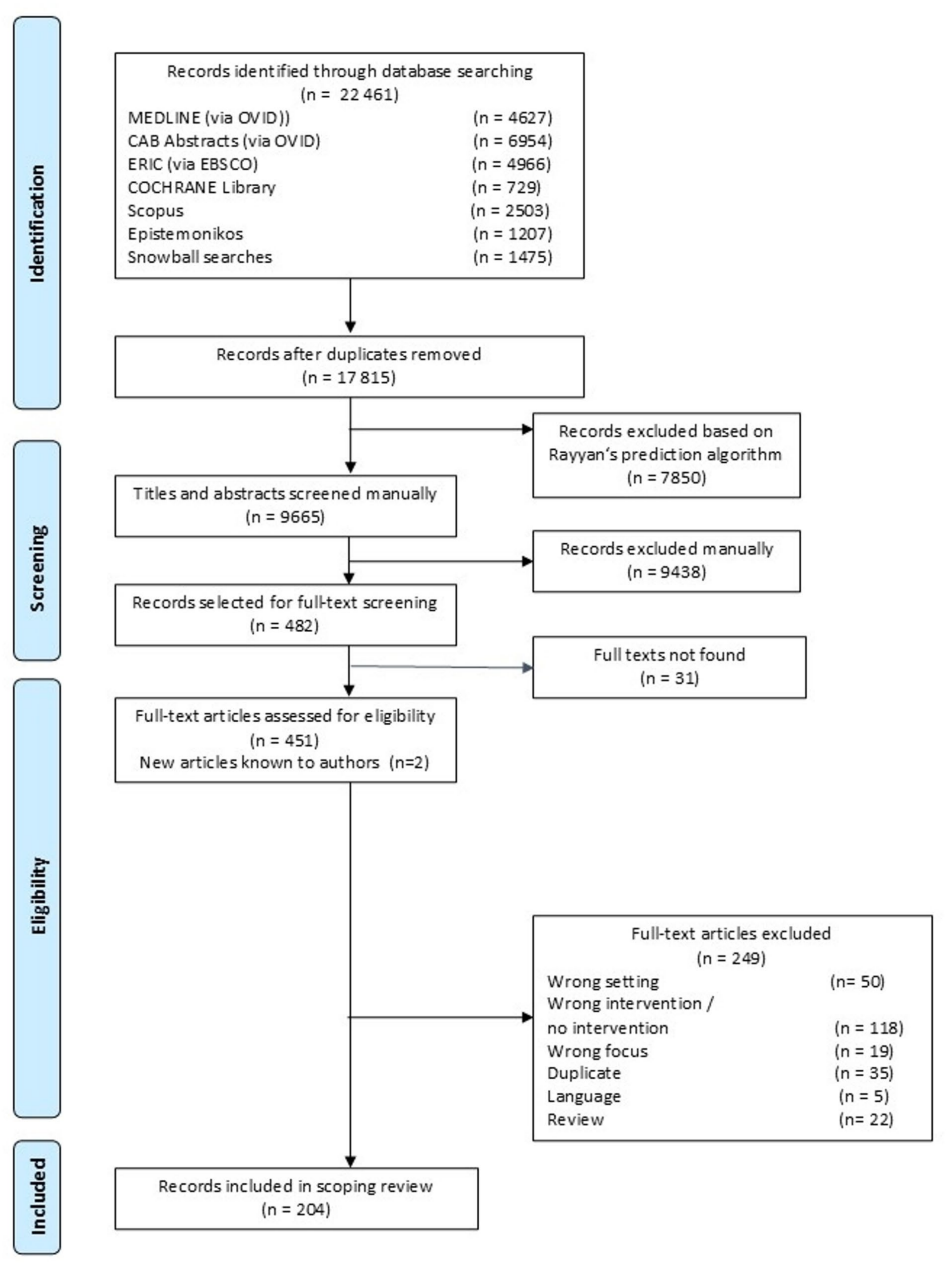
PRISMA Flow Chart

The database and snowballing searches yielded a total of 22,461 records before de-duplication and 17,815 after de-duplication. Of these, 8,605 records were identified through MEDLINE, Scopus, and the snowballing searches, and were manually screened in full. We then applied Rayyan’s prediction algorithm to the remaining 9,210 records identified through CAB Abstracts, Epistemonikos, Cochrane Library, and ERIC, excluding 7,850 records that received a star rating of below 2.5, leaving another 1,360 records with a star rating of 2.5 or higher to be screened manually. In total, we excluded 17,333 records at title and abstract screening stage, leaving 482 records for full-text screening, of which we included 204 records reporting on 206 studies in our review (two records reported on two studies each [27, 28]). Among the studies excluded at the full-text screening stage were five studies in Korean and Chinese, for which we were unable to arrange translation. An exemplary list of studies excluded at this stage with reasons for exclusion is provided in table s2 in supplementary file 1. The search and screening process, including the number of studies retrieved through each search method, and the number of studies excluded for specific reasons during full-text screening, is depicted in the PRISMA flow diagram in Fig. 1.

### Characteristics of included studies

A full list of included studies with key study characteristics is provided in supplementary file 1. A spreadsheet allowing for filtering by study characteristics is available online at https://osf.io/ykv3n. In the following paragraphs, we present a short overview of study characteristics.

#### Study type, study design, country focus, year of publication, and setting

**Fig. 2:**
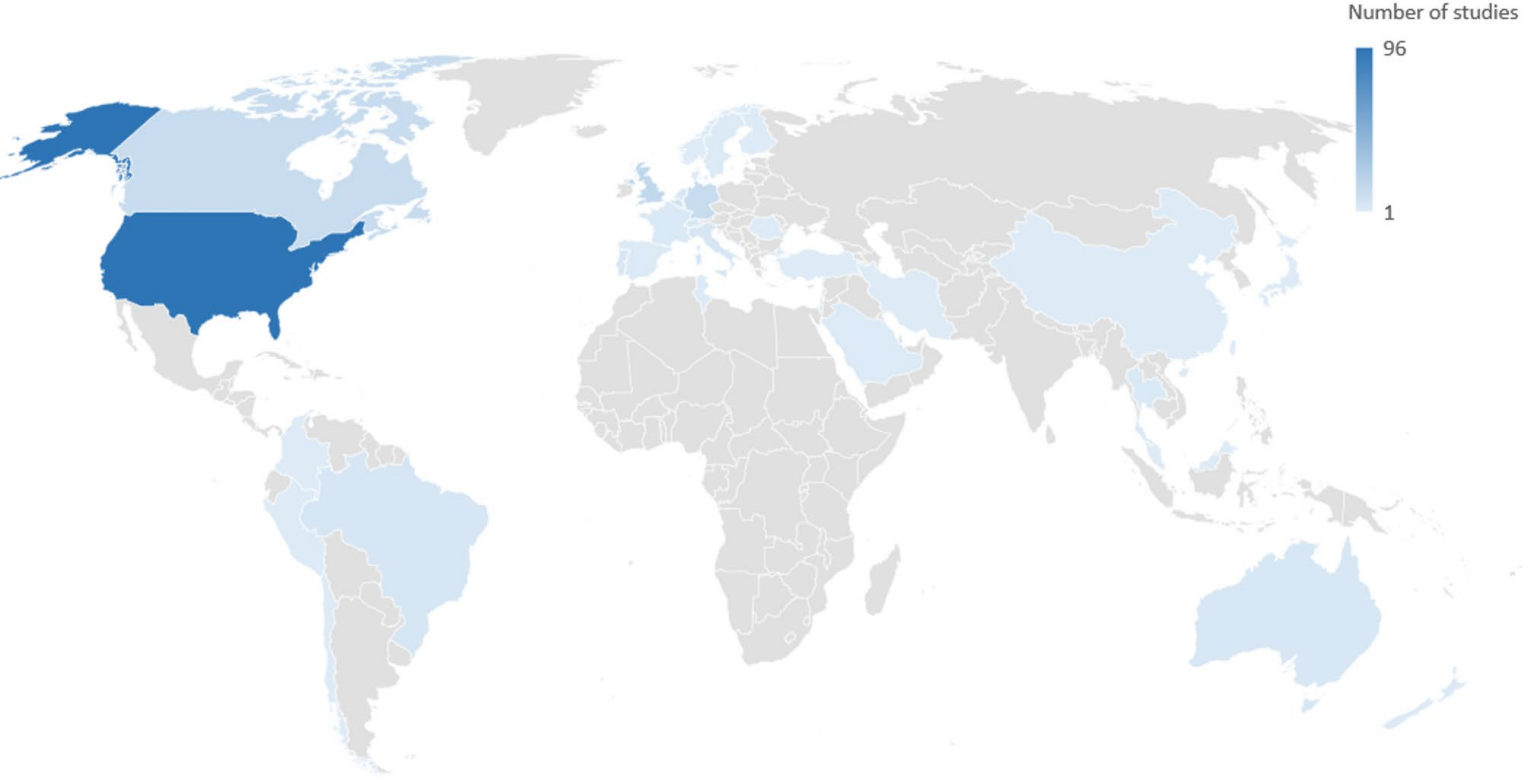
Number of studies per country

**Fig. 3:**
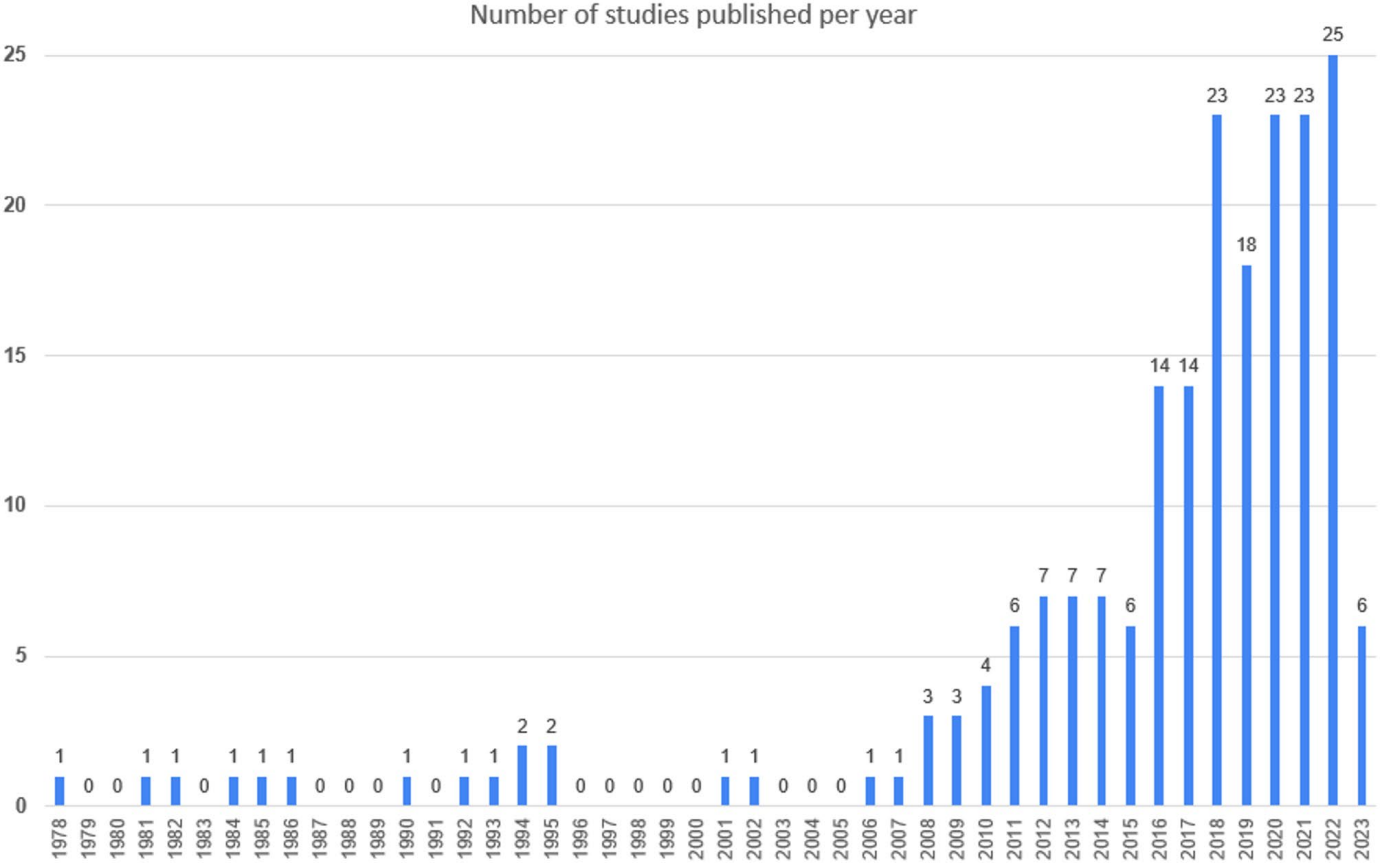
Number of studies published per year. Note that searches were conducted in June 2023, i.e. studies published in 2023 are not fully captured

The most common study design was quasi-experimental (including before-and-after-studies, and more sophisticated designs such as interrupted time series studies). Among the included studies, 143 studies (69% of the total) used quasi-experimental designs, followed by cross-sectional designs (*n* = 31, 15%), randomized controlled trials (*n* = 16, 8%), qualitative studies (*n* = 13, 6%), and modeling studies (*n* = 3, 1%) (see Table 1). Of the quasi-experimental and cross-sectional studies, 16 (8%) were mixed-methods and also had a qualitative component. The majority of studies were from North America (*n* = 109, 53%) or Europe (*n* = 70, 34%), and only few (*n* = 25, 13%) from other regions. Regarding countries, most studies were conducted in the US (*n* = 96, 47%), the UK (*n* = 16, 8%), as well as Canada and Germany (*n* = 13, 6%, each) (see Table 1; Fig. 2). The number of studies published per year increased markedly over time, with an average of 23 studies published per year since 2018 (see Fig. 3). Regarding the type of food service operation, three out of four studies focused on cafeterias or canteens (*n* = 157, 76%), while 21 studies (10%) focused on vending machines on campus, 17 (8%) reported on campus-wide interventions, and 11 (4%) on interventions in cafés, kiosks or in class (see Table 1).

**Table 1:** Study designs, settings, and countries of included studies

Characteristics	*n*	%
Study design		
*Quasi-experimental*	*143*	*69%*
*Cross-sectional*	*31*	*15%*
*Randomized controlled trials*	*16*	*8%*
Qualitative*	*13*	*6%*
*Modelling*	*3*	*1%*
**World region**		
*North America*	*109*	*53%*
*Europe*	*70*	*34%*
*High-income Australasia*	*9*	*4%*
*Latin America*	*8*	*4%*
*East Asia*	*3*	*1%*
*South-East Asia*	*3*	*1%*
*Middle East*	*3*	*1%*
*Africa*	*1*	*0%*
**Type of food service operation**		
*Cafeteria / canteen*	*157*	*76%*
*Vending machine*	*21*	*10%*
*Campus wide*	*17*	*8%*
*Café*	*6*	*3%*
*Kiosk*	*3*	*1%*
*In class*	*2*	*1%*

Notes: *This is the number of studies using only qualitative methodology. A further 16 studies were mixed-methods

#### Study objective

The majority of studies (*n* = 163, 79%) focused primarily on promoting healthy and sustainable dietary patterns among food service users. In comparison, 25 studies (12%) aimed at food waste reduction, and 18 studies (9%) investigated other approaches for increasing the sustainability of food service operations. On a more detailed level, the most common primary objectives were promoting healthy and sustainable diets in general (i.e. not targeting a specific food or nutrient; *n* = 78, 38%), reducing consumption of animal-based foods (*n* = 23, 11%), and increasing fruit and vegetable intake (*n* = 20, 10%) (see Table 2).

**Table 2:** Primary study objectives

Study objective	*n*	%
Promotion of healthy & sustainable diets	163	79%
*Healthy and sustainable diets in general*	*78*	*38%*
*Reduced intake of animal-based foods*	*23*	*11%*
*Increased fruit & vegetable intake*	*20*	*10%*
*Reduced calorie intake*	*12*	*6%*
*Reduced sugar intake*	*10*	*5%*
*Reduced carbon footprint*	*8*	*4%*
*Increased whole-grain intake*	*6*	*3%*
*Reduced salt intake*	*3*	*1%*
*Reduced water footprint*	*1*	*0%*
*Reduced fat intake*	*2*	*1%*
**Food waste reduction**	**25**	**12%**
**Sustainable food service operations**	**18**	**9%**
*Sourcing practices*	*9*	*4%*
*Recycling/composting*	*5*	*2%*
*Reduced packaging waste*	*3*	*1%*
*Energy efficiency*	*1*	*0%*

#### Classification according to the NOURISHING framework

We classified the approach taken by the interventions reported in the included studies using the NOURISHING Framework. The majority of studies (*n* = 151, 73%) employed a single approach, while 40 studies (19%) combined two approaches, 14 studies (7%) implemented three approaches, and one study utilized four different approaches. The most commonly used strategies were labelling (*n* = 71, 34%), increasing the availability or improving the offerings of healthy and sustainable options (*n* = 67, 33%), and interventions focused on information and awareness-raising (*n* = 45, 22%) (see Table 3).

**Table 3:** Classification of included studies by intervention approach

Intervention approach^a^	*n* (%)*	Examples
Food environment interventions		
Labelling interventions	71 (34%)	Health-related labelling: nutritional traffic light labels, calorie labelling, health score labelling, nutritional values shown next to meals. Sustainability-related labelling: carbon footprint/CO_2_ labelling, labelling with sustainability ratings.
Offer healthy and sustainable food	67 (33%)	Changing the offer towards a greater choice of healthier or more sustainable options (e.g., more healthier snack options in a vending machine, less sugar-sweetened beverages on offer, offering more vegetable side dishes in the cafeteria). Nudging interventions directing consumers towards healthier and/or more sustainable choices (e.g., changing the order of meals, making certain foods more salient).
Economic tools	21 (10%)	Price reduction for healthier and/or more sustainable options (e.g. the vegetarian option, salad instead of fries, low sugar drinks). Price increases for less healthy and/or less sustainable options. Rewards for avoiding food waste.
Advertisement regulation	0	n.a.
Improve food quality or sustainability	28 (14%)	Changing recipes for a better nutrient profile, replacing animal-based proteins with plant-based proteins, using less salt or more whole wheat flour, reducing the portion size of less healthy and/or less sustainable meal components
Other food service interventions	30 (15%)	Other changes to the food environment to reduce food waste or to promote recycling, including changes towards less plastic in the food service.
**Food system interventions**		
Food supply chain interventions	8 (4%)	Changing sourcing toward local or organic produce.
**Behavior change communication interventions**		
Information and awareness-raising interventions	45 (22%)	Posters and information campaigns on healthy eating, food waste avoidance, correct recycling, and the links between food and sustainability in general.
Counselling interventions	0 (0%)	n.a.
Education and skill-building interventions	7 (3%)	Courses for food service users on healthy and sustainable eating, training for kitchen staff on preparing appealing plant-based dishes, and minimizing food waste during meal preparation.

Note: a) classification based on the NOURISHING framework (27); some studies use more than one intervention approach; therefore, percentages add up to more than 100%

#### Length of follow-up

The length of follow-up differed considerably across studies. Most studies were of short duration, with a median follow-up period of 4 weeks (interquartile range 2–13 weeks). Only 13 studies (6%) had a follow-up of one year or longer. In 60 studies (29%) the length of follow-up was either not reported or not applicable due to the study design (e.g. cross-sectional studies or formative research focusing on proposed or potential interventions).

#### Participants

The median number of participants in the studies investigated was 352 (interquartile range 162–695). In 71 studies (34%), the number of participants was either not reported or not applicable (e.g., studies reporting the food waste at the cafeteria level or using sales data reported per food item and not per customer).

#### Outcomes

The most commonly reported types of outcomes were implementation-related outcomes, including acceptability, feasibility, implementation fidelity, uptake, and views on (potential) interventions (*n* = 107, 52%). This was followed by outcomes related to diet quality, e.g. changes in sales or intake of specific food items, as well as changes in energy or nutrient intake (*n* = 104, 51%). The third largest group were sustainability outcomes (*n* = 76, 37%), including changes towards more plant-based diets (e.g. a reduction in meat consumption) or reduction of food waste. Other outcomes were examined to a smaller extent: economic outcomes, like costs or profitability were reported by 11 studies (5%); health outcomes (including mental health outcomes like weight concerns) by 6 studies (3%).

### Evidence gap map

**Fig. 4:**
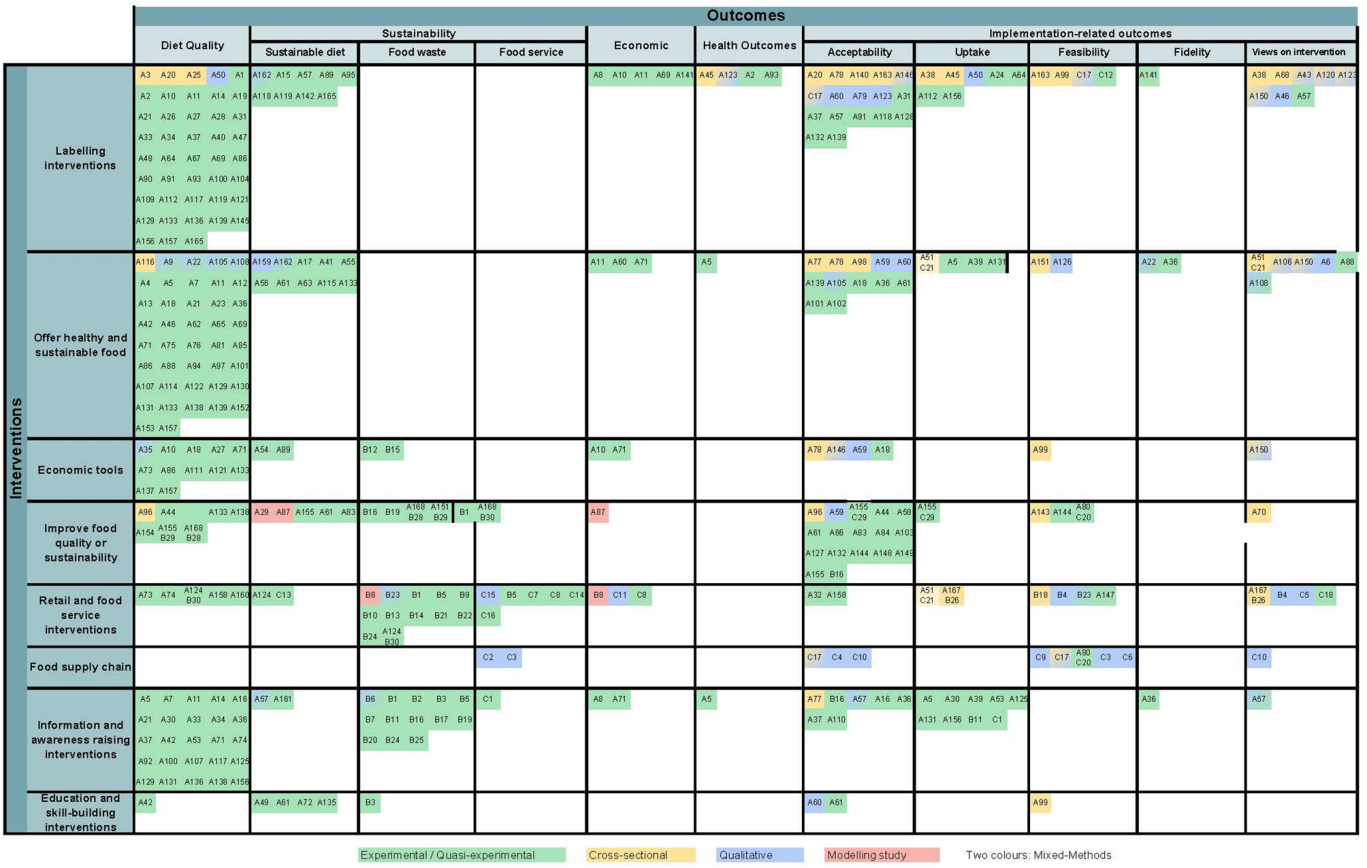
The Evidence Gap Map

The Evidence Gap Map (see Fig. 4) provides a visual summary of the interventions and outcomes explored in the included studies, along with the corresponding methods used to investigate each pairing of intervention and outcome. Each pair is also marked with a code indicating the respective study, as recorded in the list of included studies provided online at https://osf.io/ykv3n. Codes starting with ‘A’ (e.g. A1, A2, etc.) indicate studies aimed at supporting healthy and sustainable dietary patterns. ‘B’ stands for studies focused on reducing food waste, while ‘C’ represents studies targeting other aspects of improving sustainability in food service operations. By far, the largest group of intervention-outcome-pairs are represented by ‘diet quality outcomes’ for ‘Labelling interventions’ and for ‘Offering healthy and sustainable food’, with 42 (20%) each. ‘Acceptability’ as an outcome variable was studied across all intervention types, particularly for ‘Labelling interventions’ (*n* = 17, 8%) and for ‘Information and awareness-raising interventions’ (*n* = 16, 8%). While most intervention-outcome combinations are represented in the table, there are some notable gaps. ‘Labelling interventions’ were the only type of intervention evaluated for mental health outcomes. Also, only one health outcome was reported for ‘Offering healthy and sustainable food’ and ‘Information and awareness-raising interventions’, both from the same study [29].

## Discussion

### Summary of main findings

A wide variety of approaches exists for promoting health and sustainability in university and college food service settings. These include health and sustainability labels, improving the availability of healthy and sustainable options, nudging interventions to encourage healthier and more sustainable choices, economic instruments such as price adjustments, and communication measures to induce behavior change. We identified 206 studies examining such interventions, with the most common study designs being quasi-experimental, cross-sectional, and qualitative. The majority of studies were conducted in a small number of countries, in particular the US, Canada, the UK, and Germany. Implementation- and diet-related outcomes were the most frequently assessed. Studies were typically of short duration. The intervention types most commonly examined in the existing literature were labelling interventions, interventions aimed at increasing offerings of healthy and sustainable foods, and information and awareness-raising interventions. The most common outcome type were measures of dietary quality (e.g. the amount of fruit and vegetables consumed), and the acceptability of interventions.

### Comparison with similar studies and implications for research

Several reviews on related topics have been published to date. A scoping review of food and nutrition interventions in post-secondary educational facilities, published in 2021, identified 38 publications based on a narrower search in three databases, restricted to articles published in English between 2015 and 2019 [8]. They concluded that such interventions have the potential to positively impact health and sustainability, and highlighted the need for methodologically more robust evaluations that consider both human and planetary health. In contrast to our review, the 2021 scoping review did not identify any study examining both health and sustainability outcomes and included only a small number of studies on sustainability [8]. Notably, a substantial part of the studies addressing this dual focus in our review were published after 2019, highlighting the evolution of the field towards a more integrated approach. Additionally, a recent systematic review of interventions aimed at improving sustainability in food service operations—such as reducing food waste, minimizing single-use-items and packaging waste, and promoting sustainable diets—reported significant pro-environmental changes in consumer expectations, behaviors, as well as cognitive and affective attitudes [30].

Future research should focus on examining both health and sustainability jointly, given their shared importance for human and planetary health. Implementation-related outcomes (such as costs, acceptability, perceived feasibility, etc.) were examined in a substantial number of studies. Given the fact that these aspects can be crucial for sustainability and the scaling-up of interventions, these outcomes should be maintained in future investigations. Mental health outcomes, such as the potential effects of dietary interventions on eating and body image disorders (e.g. orthorexia and anorexia), were only examined in a small number of labelling studies, despite the high prevalence of these disorders among young adults. Given the magnitude of the challenges faced by the food system globally, studies on how to improve diets across the population of interest (rather than just among individuals particularly open to change) should also be a priority. These research gaps should be addressed in future studies [31].

Most studies were of short duration, with a median length of follow-up of 4 weeks. Longer follow-up durations are needed to capture the sustained impacts of interventions and account for potential changes over time. Most studies used relatively simple, less trustworthy study designs with no or only limited controls for confounding (the most common were uncontrolled before-after studies). While randomized controlled trials (RCTs) may, in many cases, not be feasible, more sophisticated quasi-experimental methods such as interrupted time series (ITS) studies may be and should be used more often [32]. Reliance on aggregated data, such as sales figures, limits the ability to examine differential effects across population subgroups, such as vegetarians versus non-vegetarians. One of the few studies that conducted such analysis found that a price reduction on vegetarian dishes combined with a price increase on meat dishes primarily changed the consumption behavior of individuals who had already eaten limited amounts of meat prior to the intervention. In contrast, it had little effect on those in the “least vegetarian” quartile, and thus on individuals who had rarely ordered vegetarian or vegan meals before [33]. These findings emphasize the need for more detailed and stratified analyses to better understand the effects of interventions and thus to be able to design interventions that more effectively target certain groups.

### Implications for policy and practice

A large variety of approaches for promoting health and sustainability in the setting of university and college food services, such as cafeterias and canteens, exist. These include, among others, health and sustainability labels; increased and improved offerings of healthy and sustainable options; nudging interventions to facilitate healthier and more sustainable choices; economic instruments such as price increases or rebates; and a variety of behavior change communication measures. While the evidence base for effective interventions is growing, important research gaps remain, as outlined above. To address these, implementation should be accompanied by methodologically sound evaluations, such as high-quality ITS studies or cluster-RCTs. Collaboration between food service practitioners, university administrators, and researchers can facilitate this process. Process evaluation can also be helpful when comparing study results, thereby improving understanding of which interventions work best in specific settings [34].

### Strengths and limitations

Our review has several important strengths. We used state-of-the-art scoping review methodology as recommended by the JBI Handbook and the PRISMA-ScR reporting guideline, which are considered gold standards in the field [17, 18]. The review followed a pre-registered protocol [19], with any deviations transparently documented in supplementary file 1. Comprehensive literature searches were conducted in six databases by an experienced information specialist. We also implemented several quality assurance procedures to ensure consistency among review authors and to minimize the risk of errors, such as a piloting of key procedures, the use of internal guidance documents, and regular team meetings.

Despite its strengths, this review also has some limitations. We did not double-screen all records, but only those marked as “unclear” by the first review author. We did not screen all references manually, but partly relied on an artificial intelligence-based prediction algorithm not yet validated for food systems research. While the use of such artificial intelligence (AI) tools may enable screening of a larger volume of studies and increase the efficiency of the review process, it may introduce bias, and poses ethical challenges [35, 36]. Data extraction was done by a single review author, and only double-checked for uncertain cases and a random subset (10%). We did not detect any discrepancies in this subset, but we cannot rule out reviewer errors. We assessed only the reported direction of effects, without evaluating effect sizes, and we did not conduct a quality appraisal of included studies, and did not assess the outcome-specific certainty of evidence, as common for scoping reviews. While we assessed the length of follow-up, we did not extract data on the duration of the intervention. Lastly, terminology inconsistencies describing campus food service operations across countries posed a challenge. While we excluded studies that were clearly about privately-operated restaurants or cafes that were located on or around campus without an affiliation to a university or college, we acknowledge that the distinction between such private establishments and university food service operations may not always be clear-cut (for studies conducted in food service establishments on campus that did not mention explicitly if they were affiliated with the university or college or not, we assumed the former).

## Conclusions

A broad range of studies on interventions to promote health and sustainability in university food service settings exist. Future research should address current gaps and limitations by examining both health and sustainability outcomes, including mental health impacts, and by using more robust experimental and quasi-experimental study designs with longer follow-up periods. Implementation efforts should be accompanied by methodologically sound evaluations using appropriate study designs with the ability to control for confounding (e.g. controlled interrupted time series studies). Such evaluations may allow for evidence-based tailoring and adjustments while also improving the overall evidence base.

## Supplementary Information

Below is the link to the electronic supplementary material.

Supplementary Material 1

Supplementary Material 2

## Data Availability

All data described in the manuscript is published alongside the article as supplementary material or is available on the Open Science Framework at https://osf.io/ykv3n/.
